# Clothing identification via deep learning: forensic applications

**DOI:** 10.1080/20961790.2018.1526251

**Published:** 2018-10-17

**Authors:** Marianna Bedeli, Zeno Geradts, Erwin van Eijk

**Affiliations:** a University of Amsterdam, Amsterdam, The Netherlands;; b Netherlands Forensic Institute (NFI), Den Hague, The Netherlands

**Keywords:** Forensic sciences, digital forensic, clothing classification, attribute identification systems, deep learning, large scale dataset, surveillance camera.

## Abstract

Attribute-based identification systems are essential for forensic investigations because they help in identifying individuals. An item such as clothing is a visual attribute because it can usually be used to describe people. The method proposed in this article aims to identify people based on the visual information derived from their attire. Deep learning is used to train the computer to classify images based on clothing content. We first demonstrate clothing classification using a large scale dataset, where the proposed model performs relatively poorly. Then, we use clothing classification on a dataset containing popular logos and famous brand images. The results show that the model correctly classifies most of the test images with a success rate that is higher than 70%. Finally, we evaluate clothing classification using footage from surveillance cameras. The system performs well on this dataset, labelling 70% of the test images correctly.

## Introduction

Rapid technological progress over the last couple of decades has allowed forensic professionals to leverage advanced tools and techniques for automatic facial recognition to identify individuals. Such techniques benefit forensic investigations by detecting face images that can be linked back to the person of interest [1].

Nevertheless, even today, facial recognition techniques are technically demanding, difficult to apply in practice, and do not always produce the desired results when facial images are taken under non-ideal conditions [2], especially when the video quality is not sufficiently high or the face is covered. To tackle this problem and increase the effectiveness and accuracy of identifying persons of interest, scientists have pursued research aimed at developing biometric methods that rely on features such as fingerprints, palm patterns and voice [3]. However, when this biometric evidence is absent, a common visual aid to facilitate the process of identifying individuals tends to be clothing.

Traditionally, attire has often served as significant visual evidence, used not only to describe people, but also to define personal information such as age, sex and social status [4]. Interestingly, even nowadays, clothing remains an important factor for describing people in detail. Recent research highlights the importance of clothing for targeting suspects or identifying missing persons [5]. In addition, Chen et al. [5] state that clothing recognition is very effective when it comes to real-time videos such as those from surveillance cameras, where clothing details can be used to pinpoint suspected criminals or missing individuals.

For instance, in the Boston terrorist attack, which took place in April 2013, clothing recognition played a key role in identifying the suspects. In the immediate aftermath of the attack, the police conducted an investigation based on numerous victim testimonies to ultimately identify the two suspected criminals as the brothers Tamerlan and Dzhokhar Tsarnaev. During the investigation, local police provided the public with a detailed description of the clothes worn by the suspected criminals, asking witnesses to contact the police if they had seen someone that matched the description. Taking this event into account, Feris et al. [6] postulated that the investigation could have been even more efficient if the police had a search system specifically designed for attribute (e.g. clothing) recognition using existing surveillance footage.

## Forensic relevance

As discussed above, attribute based identification systems are very important when it comes to identifying persons who have committed criminal activities such as terrorist attacks. The problem of identifying missing persons is also relevant. Feris et al. [6] specifically note that clothing identification could help parents find their children in crowded places simply by performing an automated search based on specific attributes using surveillance cameras. For example, if a missing child wore a red coat and carried blue backpack, the automated system would only search for matches based on these details.

Taking the above into consideration, the aim of this project is to create a tool capable of detecting and classifying clothing attributes from images. Such information could be of special importance to forensic investigations because forensic professionals are able to collect evidence about attackers or missing persons from a well-established system of digital sources. Therefore, the proposed method improves clothing identification to facilitate the investigation process.

Finally, personal traits such as clothing characteristics could complement facial recognition techniques [6] and thus the questioned model will be a potential assistance in the process of human identification.

## Related work

At present, clothing recognition is widely accepted as one of the most sophisticated techniques for visual investigation, attracting attention from numerous deep learning researchers [4,7,8]. Deep learning is a subclass of machine learning that uses artificial neural networks for tasks such as visual recognition. Unlike early neural networks, deep learning neural networks contain more than one hidden layer [9]. A neural network is a conceptual model of the human brain that can be trained by a computer to see the world is a manner similar to that of humans, and by “see”, we really mean “understand” [10]. To apply the deep learning framework, most researchers use convolutional neural networks (also known as CNNs or ConvNets), a popular deep learning technique for analysing visual imagery [11].

Research on convolutional neural network-based clothing recognition often focuses on fashion classifications or detecting clothing by processing surveillance camera footage. More precisely, Liu et al. [12] introduced a robust clothing recognition and retrieval based on classification and a large-scale dataset. Similarly, Lao et al. [4] examined and presented clothing attribute and type classification. However, real-time clothing identification from surveillance videos remains highly challenging because of the difficulties involved in achieving reliable clothing detection and representation. Yang et al. [7] partially addressed this when they proposed a system to track clothing attributes in real time videos.

Nevertheless, forensic professionals still lack reliable tools for clothing identification, creating a gap in the investigation process. The main objective of this study is to close it by using deep learning to detect and classify clothing attributes. Images of various outfits are used as data to train the computer to identify people based on clothing attributes.

Specifically, the evaluation in this study is divided into three main parts. The first part consists of clothing classification in good quality images and the second part investigates attribute classification. Unlike previous work that mostly relies on the recognition of patterns such as stripes or dots, this study focuses on logo identification. The last part involves clothing classification in unconstrained images, and its aim is to evaluate whether the system can be trained using poor quality samples to perform classification. The overall aim is to evaluate the performance of the system given the results of each experiment.

## Dataset

Before describing the proposed method, we outline the steps involved in sourcing images for the three datasets used in this study.

This study first uses the Deep Fashion database, compiled by Liu et al. [12], to train the model. The dataset contains 278 461 images divided into 5 412 categories. This dataset is particularly useful because it also offers rich clothing information and fine attributes labels.

**Figure 1.**
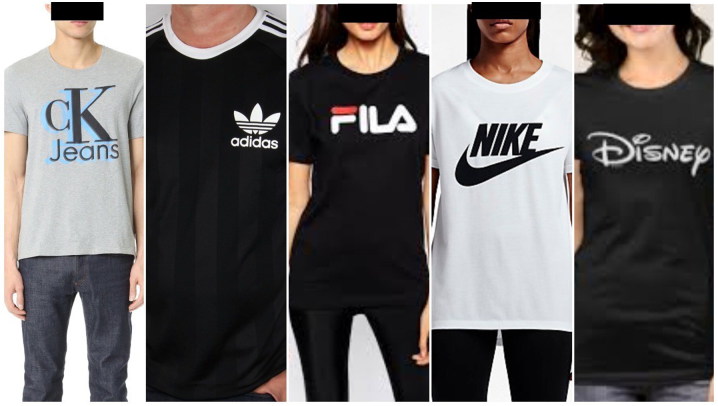
Example images from the Logo dataset. (With permission)

Once the model has been sufficiently trained, this study uses a second dataset called the Logo dataset, which contains even more detailed clothing characteristics, to test the performance of the proposed model. Unlike the Deep Fashion dataset, this second dataset focuses specifically on details such as popular logos and brand images. Because previous studies have most commonly experimented with clothing patterns such as stripes and checkered textures, the second dataset was built entirely manually, sourcing images directly from the Internet. The dataset in question contains 5 549 images broken down into 70 categories (Figure 1).

**Figure 2.**
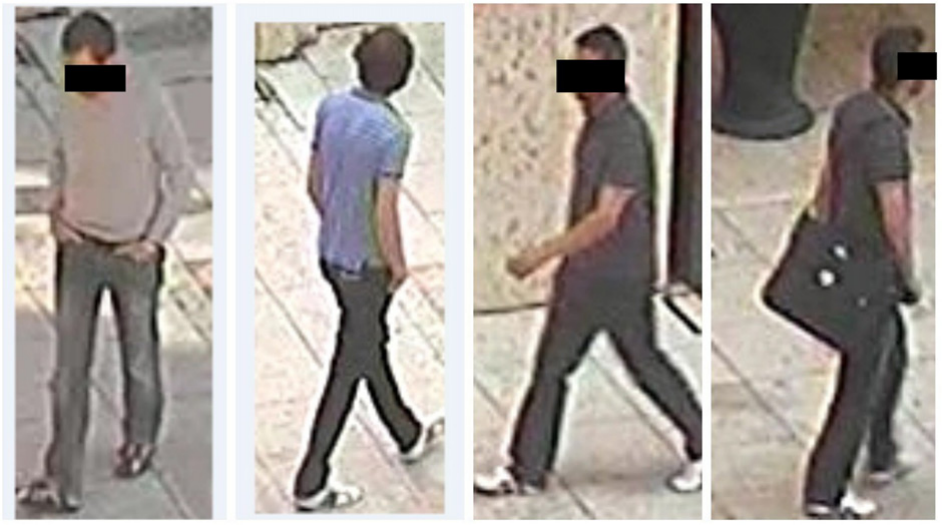
Example images from the Surveillance dataset. (With permission)

Finally, this research used a third dataset called the Surveillance dataset, which consists of images obtained by surveillance cameras to evaluate the proposed system. This is because, in the footage obtained during an investigation, most images are not taken in ideal conditions with the right pose. Therefore, the system was trained to perform classification on images with low resolution and poor quality. The evaluation used a part of the 3DPeS dataset [13]. This dataset is especially suitable because it provides different pedestrian poses and angles. This is very important for the training phase given the low quality of the data. The dataset in question includes 2 277 images broken down into four categories (Figure 2).

**Figure 3.**
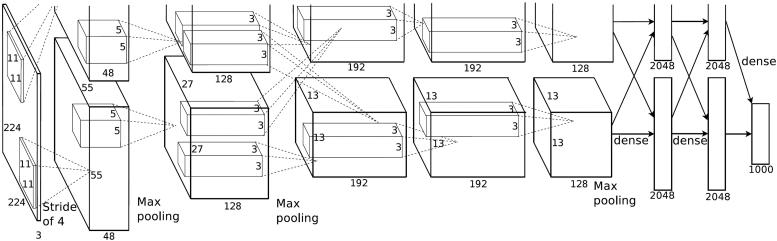
AlexNet architecture [15]. (With permission)

### Image collection

One difficulty associated with manually building datasets is devising a strategy to collect images in quantities large enough to provide the model with a sufficient number of samples. Fortunately, online clothing stores offer a rich collection of categorized clothing images.

For this reason, the images for the second dataset were primarily taken from Google Images as well as the popular ecommerce website Amazon, which features thousands of brand and logo images already organized in alphabetical order. Note that the collected images were limited to upper-body clothing because logos rarely appear on lower-body attire.

To collect a sufficient number of images within a practical amount of time from Google, custom computer scripts were used to collect them in bulk. For instance, one script scraped images based on query keywords selected by the user such as type of clothing (i.e. T-shirt and jacket) and brand (i.e. Adidas, Nike or Puma). The benefit of this method is that it searches and saves images entirely automatically; however, it often includes random unrelated images from the scraped webpage.

After the images were gathered from the Internet, data were manually cleaned to filter out all unrelated images. This was a crucial step because some of the images were not suitable given their low resolution, poor quality or irrelevant nature.

## Method

We next describe the evaluation process as well as the techniques and methods it uses.

### Architecture

To run the experiments, NVIDIA’s deep learning system DIGITS was used to classify clothing in the datasets. The system was trained on the AlexNet architecture, using ImageNet’s pre-trained weights, for both the Deep Fashion and Logo datasets. ImageNet includes many classes that are irrelevant to clothing characteristics. However, the pre-trained ImageNet model has been shown to be successful even for unrelated datasets [4]. Therefore, the default AlexNet architecture was used for these experiments.

AlexNet (Figure 3) is a convolutional neural network architecture and consists of eight learning, five convolutional, and three fully connected layers [14]. Usually, the convolutional layers include a convolution step, pooling step, and activation functions. Specifically, the convolution step is a set of learnable filters that are responsible for extracting features from the input image. They do so simply by applying element-wise multiplication to the pixels of the input image, producing the feature map.

**Figure 4.**
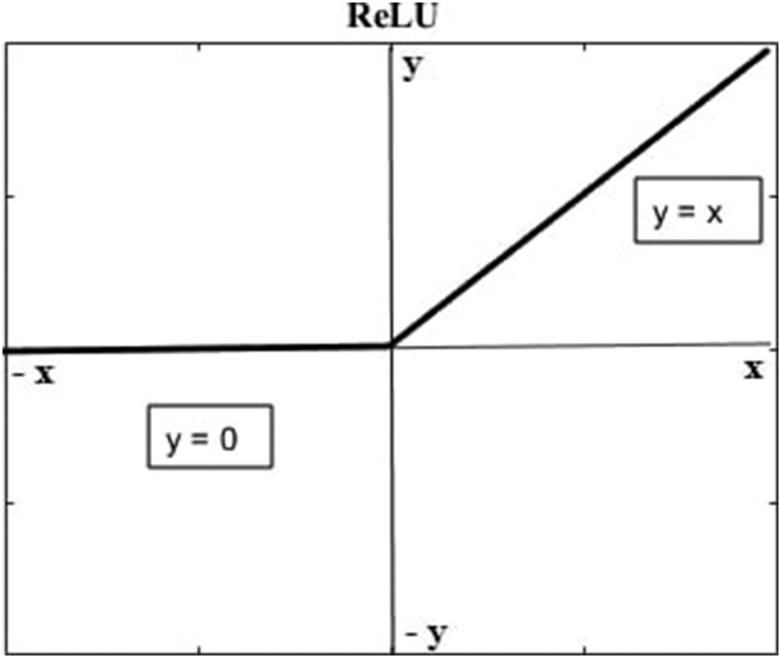
ReLU function: For negative input values, the output lies on the *x*–axis and for positive input values, it lies on the diagonal (*y* =* x*). (With permission)

The Rectified Linear Unit (ReLU) is an activation function that is used to introduce nonlinearity into the network. Nonlinearity is crucial for neural networks because it enables them to understand the real notion of depth and therefore maintain their learning power as the data are processed by deeper hidden layers. As depicted in Figure 4, ReLU is defined as *f*(*x*) = max(0, *x*), so it effectively replaces all negative values in matrix *X* with zero and retains all non-negative values [15].

**Figure 5.**
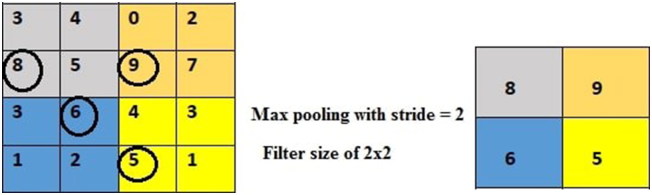
Max pooling with a filter size of 2 × 2 and stride of 2. The right matrix outputs the maximum value of each of the subregions of the input matrix.

In addition, pooling (max pooling in this case) is used to reduce the spatial size and complexity of the network. It does so by simply outputting the maximum element from a matrix. As shown in Figure 5, a filter is applied to the input matrix that moves across every 2 × 2 subregion, outputting the maximum element from each one. The number of pixels that the filter moves each time is referred as its stride [16].

**Figure 6.**
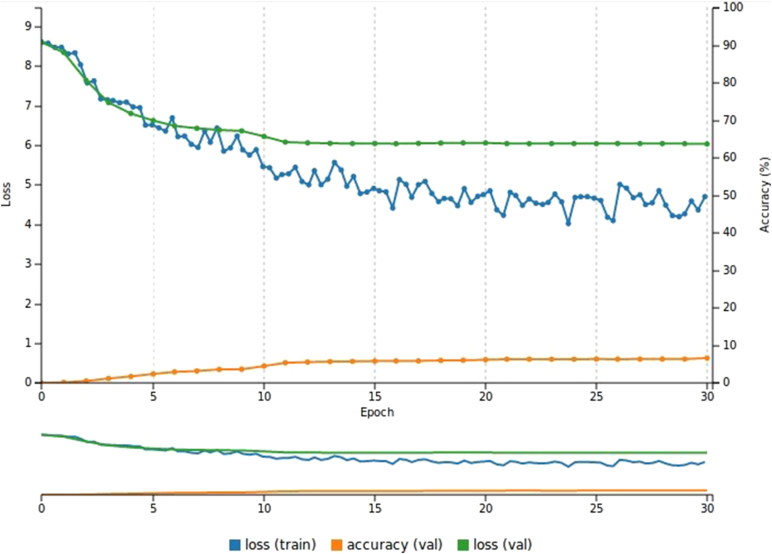
Performance of the model on the Deep Fashion dataset (30 epochs). The accuracy of the model is low (6.6%) while the validation loss is 6.03.

At the end of the neural network architecture, there are the fully connected layers, where every neuron is fully connected to the neurons of the next level. The fully connected layers are essential for the network because they use the output (usually called the ‘high-level features’) from the previous layers to classify the input image [17].

For the third dataset (the images from the surveillance cameras) LeNet architecture was used to train the system. However, a slightly different model from the original LeNet architecture was applied, using ReLU instead of sigmoid activations for the neurons [18]. The reason behind this is that ReLU has been shown to be more efficient and faster than sigmoid in deep networks [14]. The LeNet network consists of two convolutional layers and two fully connected layers.

### Parameters

#### Kernel size

Each convolutional layer includes parameters such as the number of outputs and kernel size. The number of outputs is the number of learnable filters that are applied in the layers, while the kernel size refers to the height and the width of each filter. These two parameters are essential for the network because they enable key features in the input image to be detected.

In the AlexNet architecture we used the default kernel size; however, in LeNet, we changed the kernel size to two instead of the default five. A larger kernel size increases the likelihood that details will be ignored and essential features skipped. On the contrary, when the size of the kernel is small, this can lead to the retention of more information [19]. Especially when the model was trained on the third dataset with the unconstrained images, it was very important for the algorithms to have access to more specific information.

#### Batch size, iterations, and epochs

After choosing the architecture of the networks, it was necessary to determine the batch size, number of iterations, and number of epochs used to train the networks. The batch size is the number of training set samples that are propagated through the network each time. In addition, the number of iterations refers to the number of times a batch is propagated through the backpropagation algorithm, which is responsible for updating of the weights of the neurons to minimize the error of each output neuron [18]. Finally, the number of epochs describes how many times the algorithm processes the entire dataset. Therefore, an epoch is completed successfully only after the algorithm has processed the entire dataset [20].

For the Deep Fashion dataset, we used batches of 128 images for training. The total number of training samples was 167 052, which, divided by 128, resulted in approximately 1 305 iterations per epoch. Thirty epochs were used to train the system, resulting in 39 150 iterations.

The solver was stochastic gradient descent. When using this solver, it is advisable to initialize the learning rate to about 0.01 [14]. This initial learning rate was decreased by a factor of 10 after every 13 050 iterations (10 epochs). In addition, the model was tested on the validation set after every 1 305 iterations (one epoch).

For the Logo dataset, we trained the model again in batches of 128. The total number of training samples was 3 328 images, which resulted in 26 iterations per epoch. One hundred and eighty epochs were used to train the system, leading to 4 680 iterations. The initial learning rate was 0.01 and decreased by a factor of 10 after every 1 560 iterations (60 epochs). For every 26 iterations (one epoch), the model was tested on the validation set.

For the Surveillance dataset, we trained the model in batches of 64. The training set was 768 images, resulting in 12 iterations per epoch. Thirty epochs were chosen; therefore, the total number of iterations used was 360. The learning rate was set to 0.01, decreased by a factor of 10 after every 120 iterations (10 epochs). In addition, after every 12 iterations (one epoch), the model was tested on the validation set.

#### Training, validation, and test sets

The three datasets were each split into three subsets: a training set, validation set and test set. Sixty percent of all images were assigned to the training set; the validation and test sets were both assigned 20% of the remaining datasets [21].

The training set is the largest and is designed to teach the network how to adjust the weights, assigning them coefficients according to their likelihood of minimizing errors in the results. Because this part of the process is particularly laborious, more than half of all the images are allocated to it.

The validation set stores the data that are used to prevent overfitting; it also allows users to check whether the error is within a certain range. The validation process is used to confirm that the network predicts well on this set. This usually does not require more than a quarter of the full image dataset.

Once the data have been trained and optimized, it is important to determine how the model performs on completely unseen real-world data: this is where the test data are used. The test data results should verify that the network accurately recognizes images. In most cases, around 20% of all images are considered enough to prove whether the model has been successfully trained. If the model struggles to deliver accurate predictions, it might be necessary to return to the training set and examine the hyperparameter settings to tune the network and the quality of data.

## Experiments

### Deep fashion dataset

For the experiments, the images of the Deep Fashion dataset were resized to 256 × 256. In addition, the image resize option was set to a half crop/half fill transformation and the output image format was JPEG because that format enables the dataset to occupy much less space. DIGITS, which is an interactive Deep Learning GPU training system, it displays a graphic based on the validation accuracy, training loss, and validation loss to report the performance of the model. The validation accuracy shows how accurate the model is at classifying images. The loss measures whether the error is within a certain range by measuring how much the actual output of the neurons diverges from the desired output; a loss close to zero means that the error has been minimized because the actual output is close to the desired output and the training of the model was successful. Additionally, DIGITS displays the top-5 classifications and the corresponding confidence values for a test image.

Figure 6 shows the performance of the model, which is less than satisfactory because the validation loss (6.03) is much higher than the validation accuracy, which is only 6.6%; this indicates that the validation set has an unacceptably high margin of error.

Although the accuracy performs poorly, the system classifies the test images within the top-5 predictions. One drawback is that the prediction accuracy remains quite low - with a success rate of less than 5%. For instance, a test image depicted a belted polka dot dress was classified correctly within the top-5 predictions but its rate was low ; to be more specific it scored 6.83% in the top-2 prediction.

We ran the same model for 30 trials (keeping the hyperparameters fixed), allowing the system to randomly split the dataset into training, validation, and test subsets each time. This procedure enables us to evaluate the performance of the model under different sets and obtain the average accuracy. More specifically, we focused on the accuracy of the top-1 prediction.

The average top-1 accuracy across all 30 trials was 8.1%, while the average top-1 accuracy for the represented model (Figure 6), was 8.6%.

### Logo dataset

For these experiments, the Logo dataset was split into two subsets. Specifically, the first subset contained clothing images with their logos and the second one consisted of images depicting only the logo itself. The purpose was to evaluate which of the two subsets would provide more accurate results when training the model. While this might seem counterintuitive, this approach helps us to gradually determine on which visual cues the neural networks tend to focus. Moreover, because attribute clothing classification (especially in the case of logos) has not been extensively covered in academia, it is paramount to investigate this topic.

Because the datasets have a small number of images, the LeNet neural network was chosen to train the system. Unfortunately, the results obtained from both subsets were less than satisfactory: a validation accuracy of 30.0% for the first subset and 48.4% for the second one.

Hence, we investigated whether combining the two subsets could deliver better results. For this purpose, we created one dataset that contained both subsets. To ensure homogeneity, the dataset deliberately included the same number of images for every class. In line with recent research, we increased the number of images per category in the merged dataset to obtain better results [22].

Because the new dataset was larger in size, the default AlexNet network architecture was used. The image size was set to 256 × 256, image resize transformation to half crop/half fill, and image format to JPEG.

**Figure 7.**
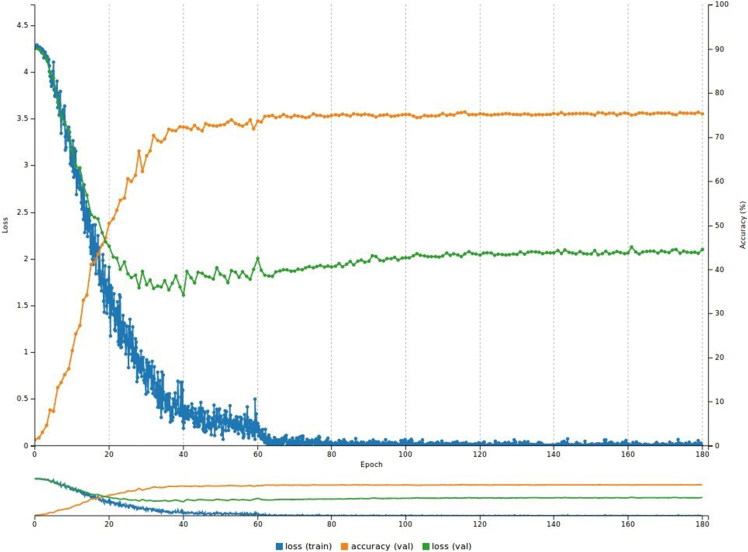
Performance of the model on the Logos dataset (180 epochs). The accuracy stabilizes at 75.4% while the validation loss is 1.9.

The accuracy reached 75.4% and the validation loss was 1.9, while the training loss reached 0.004. As Figure 7 shows, this might be a case of overfitting because the validation loss is much higher than the training loss.

**Figure 8.**
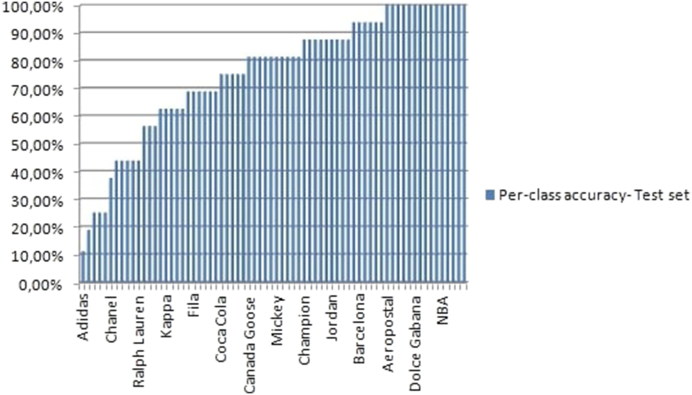
Average top-1 predictions for the 70 classes of the Logo test set.

Given the above results, we note that the AlexNet model performs much better than the LeNet model because it has more convolutional and pooling layers. Adopting the same approach as before, the average top-1 accuracy across all 30 trials was 72.9% for the test set. Figure 8 plots the average of the top-1 retrieval accuracy (74.9%) for the 70 categories based on the results from the chosen test set. Because there are 70 classes in Figure 8, Tables 1 and 2 show the categories with the five highest and five lowest top-1 accuracy rates, respectively.

**Table 1.** Five categories with the highest top-1 accuracy.

Logo	Accuracy
Aeropostal	100.00%
Banana Republic	100.00%
Chelsea	100.00%
Dolce Gabbana	100.00%
NASA	100.00%

**Table 2.** Five categories with the lowest top-1 accuracy.

Logo	Accuracy
Adidas	11.00%
Calvin Klein	25.00%
Vans	25.00%
Chanel	37.50%
Giorgio Armani	43.75%

### Surveillance dataset

For the third dataset, the LeNet network architecture was used because the Surveillance dataset is small. The image size was set to 32 × 32, the image resize transformation to half crop/half fill, and the image format was JPEG.

**Figure 9.**
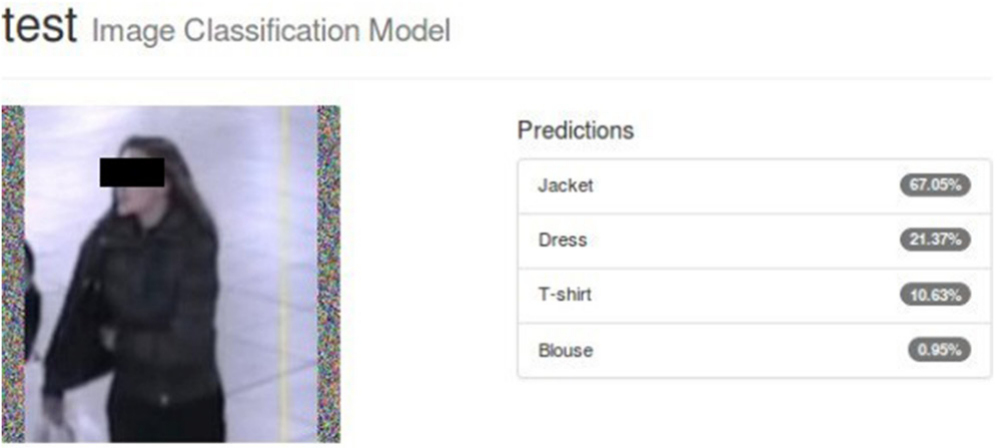
Performance of the model on the Surveillance dataset (30 epochs). The accuracy plateaus at 75.3% while the validation loss is 0.72.

**Figure 10.**
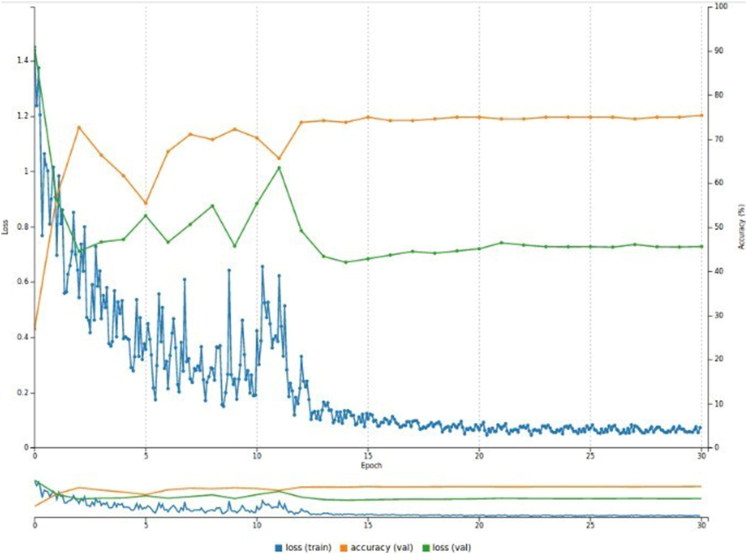
Top-4 classifications and their corresponding confidence values for a test image from the Surveillance dataset. The model correctly labelled the test images in the top-1 predicted category with a score of 67.05%.

**Figure 11.**
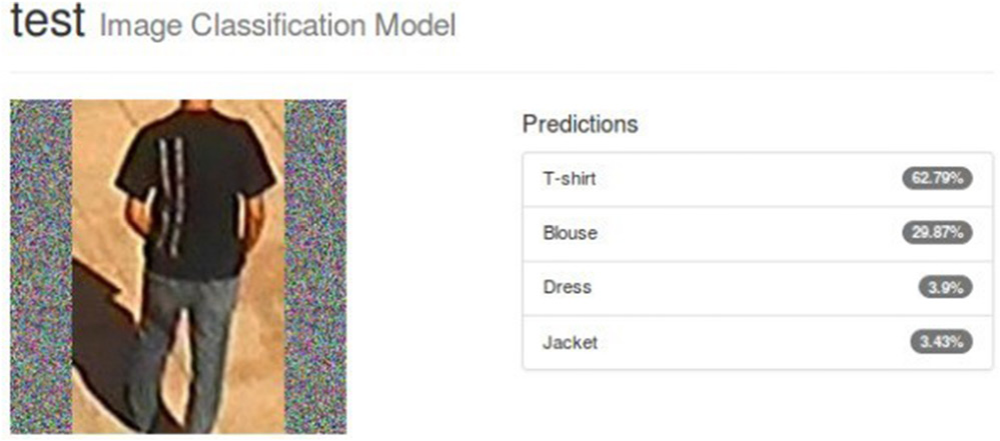
Top-4 classifications with their corresponding confidence values for an example test image from the Surveillance dataset. The model classified the test image correctly with a prediction score of 62.79%.

As depicted in Figure 9, the accuracy reached 75.3% and the validation loss was 0.72, while the training loss reached 0.07. Additionally, Figures 10 and 11 show the predictions for two test images.

**Figure 12.**
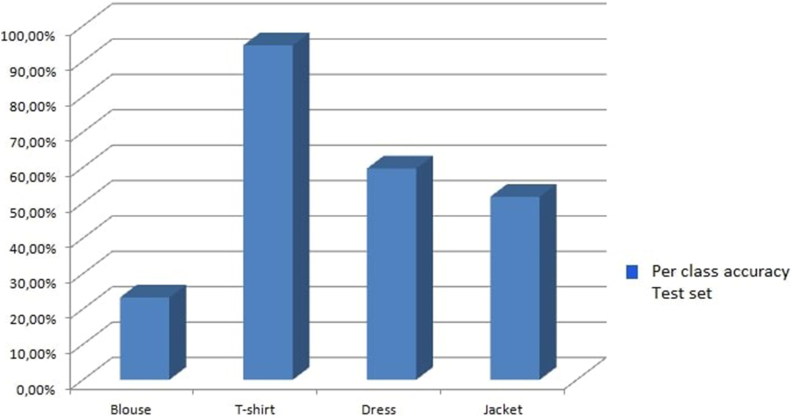
Average top-1 predictions for the four classes of the Surveillance test set.

The average top-1 accuracy across all 30 trials was 70.9% for the test set. Figure 12 plots the average top-1 accuracy for the four categories based on the results we obtained from one of the test sets, which yielded an average accuracy of 72.5%.

## Discussion

For the Deep Fashion dataset, the results demonstrate that the system’s accuracy was not satisfactory. However, the model was trained on a dataset that included more than 5 000 categories. Hence, a low accuracy does not necessarily imply that the system did not work successfully. This might have resulted because there were many classes inside the dataset and the details of these classes were too rich for even a human to spot the differences easily. To be more specific, the model was able to classify the images based on their content (e.g. a dress or T-shirt) but it failed to recognize items such as a dotted belt or stripped pocket and assign them with the correct label; for example, classifying a test image as a dress with a dotted belt. In addition, the low accuracy can partially be explained by the fact that the Deep Fashion dataset does not include homogeneity in its classes. Finally, it might be the case that the data are not appropriate for the task.

For the Logo dataset, the percentage of the accuracy fluctuated depending on the content of the test image. To be more specific, test images depicting a person wearing the clothing yielded a lower accuracy than test images that showed only the garment itself. One explanation for these results is that when the dataset contained mostly images with only the clothing itself, the system was better able to recognize these particular images.

For the Surveillance dataset, the performance of this model is satisfactory despite the poor quality of the images. The highest percentage of accuracy was achieved for the T-shirt class while the lowest was yielded for the blouse class. T-shirts and blouses share similar visual characteristics, making it difficult for the system to identify the sleeve in the test images and therefore mislabelling classifications. The dress class had the second highest accuracy rate (as expected) because the dress has more distinguishable features, making it easier for the model to identify it.

Finally, the loss during the validation phase was much higher than the loss during the training phase for all three experiments, as displayed in Figures 6, 7, and 9. There are two likely explanations for this phenomenon. The first is that the data are not appropriate, meaning that either the quality of the images is not good enough or the quantity of images is not sufficient to train the system successfully. The second reason might be that the network is not well-suited to the data, meaning that the parameter values within the neural network are too high or low. This can be solved with further experiments to fine-tune the network.

## Conclusion

In this study, a model was trained not only to classify clothing within good quality images, but also to perform well when using low resolution images. The accuracy of the model reached approximately 75% while the average top-1 prediction was around 70%, which is satisfactory performance given the low quality of the images.

Apart from the clothing classification, we also trained the system to distinguish more specific attributes such as logos. The results were satisfactory; the model achieved an accuracy of approximately 75%, with an average top-1 prediction close to 73%. The diversity of data that have been used to train the system under different circumstances makes this study relevant and innovative.

For future research, it is highly recommended the three datasets to be merged into one, creating a very large clothing dataset. The clothing dataset could be used either for large-scale classification or, combined with a dataset of facial attributes, for the process of human identification.

Developing a tool capable of applying object detection and, whenever a human is located, comparing faces and clothing attributes, would be extremely advantageous for forensic professionals. Especially in real-time situations, this tool could be used in automated search systems to enable forensic professionals to detect and identify suspected criminals or missing persons.
